# Choices of immediate open access and the relationship to journal ranking and publish-and-read deals

**DOI:** 10.3389/frma.2022.943932

**Published:** 2022-10-20

**Authors:** Lars Wenaas

**Affiliations:** TIK Centre for Technology, Innovation and Culture, University of Oslo, Oslo, Norway

**Keywords:** open access, incentives, hybrid, gold, transformative agreements, journal ranking

## Abstract

The role of academic journals is significant in the reward system of science, which makes their rank important for the researcher's choice in deciding where to submit. The study asks how choices of immediate gold and hybrid open access are related to journal ranking and how the uptake of immediate open access is affected by transformative publish-and-read deals, pushed by recent science policy. Data consists of 186,621 articles published with a Norwegian affiliation in the period 2013–2021, all of which were published in journals ranked in a National specific ranking, on one of two levels according to their importance, prestige, and perceived quality within a discipline. The results are that researchers chose to have their articles published as hybrid two times as often in journals on the most prestigious level compared with journals on the normal level. The opposite effect was found with gold open access where publishing on the normal level was chosen three times more than on the high level. This can be explained by the absence of highly ranked gold open access journals in many disciplines. With the introduction of publish-and-read deals, hybrid open access has boosted and become a popular choice enabling the researcher to publish open access in legacy journals. The results confirm the position of journals in the reward system of science and should inform policymakers about the effects of transformative arrangements and their costs against the overall level of open access.

## Introduction

Journals and their rankings have occupied an important part in the reward system of science. Choices of where to have work published influence careers and highly prestigious journals can pave the way in research evaluation processes (Heckman and Moktan, 2020). The meritorious role of journals has led to a journal hierarchy and navigation by journal rankings, of which the most debated system is the journal impact factor (JIF) (Mingers and Leydesdorff, 2015; Osterloh and Frey, 2020). JIF is one of the most important factors for researchers when choosing a target journal (Nature Publishing Group, 2015; Blankstein and Wolff-Eisenberg, 2019; Echevarría et al., 2020; Gaston et al., 2020) and impact factors and similar metrics are widely used in decisions concerning reviews, grants, promotion, and tenure (McKiernan et al., 2019; Saenen et al., 2019). This practise takes place despite extensive warnings in charters, such as San Francisco Declaration on Research Assessment (DORA)-declaration and the Leiden manifesto (DORA, 2012; Hicks et al., 2015).

The current wiring of the reward system of science can conflict with open access, the principle that all research output should be freely available and not behind subscription paywalls. Open access is linked to academic, economic, and societal benefits, in addition to probable large cost savings (e.g., Schimmer et al., 2015; Tennant et al., 2016). Nine out of ten researchers regard open access as beneficial for their field (Dallmeier-Tiessen et al., 2011) and the principle of open access is widely supported by researchers (Zhu, 2017). Open access comes in three main tiers; gold are publications in non-subscription-based journals while green is tied to depositing articles from subscription-based journals in repositories, with dissemination mostly after a publisher-imposed embargo. Finally, there is hybrid, where single articles in subscription-based journals are made free to access by payment of a fee.

However, even though researchers are in favour of open access, surveys have shown that when they choose a journal to submit to, open access is not considered important, as opposed to the reputation of the journal (Nature Publishing Group, 2015; Blankstein and Wolff-Eisenberg, 2019). Gold open access journals are considered less attractive than subscription-based legacy journals that host the other two main types of open access: green and hybrid. This has the consequence that the reward system of science continues to be an obstacle to the uptake of gold open access (Björk, 2004, 2013). Authors have expressed concerns about the quality of gold open access publications (Nature Publishing Group, 2015) and they are reluctant to pay fees for publishing (Tenopir et al., 2017). In addition, there are concerns about predatory journals, which are fraudulent journals that publish articles with little or no peer review (Shen and Björk, 2015; Richtig et al., 2018).

The meritorious role of journals and any conflict with open access must be considered by science policy, which is credited for the relatively large growth in open access in recent years (Larivière and Sugimoto, 2018; Huang et al., 2020b). A recent debated and controversial initiative is Plan S, a policy from a consortium of research funders with a preference for immediate open access, primarily in the form of gold open access journals (cOAlition, 2018). Plan S also allows for hybrid open access publishing, by actively promoting transformative publish-and-read (PAR) deals with publishers, a type of arrangement designed to turn subscription-based journals into gold open access through the hybrid pathway, in the case of Plan S, by no later than 2024.

Plan S intends structural changes in academic publishing, but by mandating certain types of journals with certain qualities the policy also interferes with choices that are important for researchers' careers. In this respect, the strategy of PAR deals should be welcome, as they make available a range of journals compatible with open access mandates and at the same time provide the researchers at participating institutions with the option of easy and cost-free hybrid publishing in their preferred outlets.

The research objective of this study is to investigate how journal ranking relates to the uptake of gold and hybrid open access, and how this uptake is affected by PAR agreements. Previous studies have focused on the uptake of different versions of open access through comparisons (Martín-Martín et al., 2018; Piwowar et al., 2018). Also, the relationship between journal ranking and gold open access has been explored (Björk and Solomon, 2012; Solomon et al., 2013; Gunasekaran and Arunachalam, 2014; Huang et al., 2019) and whether open access can be considered beneficial for increasing journal rank (Li et al., 2018; Wei, 2020). However, these studies often take the general approach of investigating gold open access journals in comparison with other types of journals. The novel approach of the present study is to investigate choices of gold and hybrid publishing, both separate and in comparison, from the viewpoint of an author's potential journal options and their rank. Further, as far as I have found, PAR deals have not yet been empirically investigated in the literature.

Norway offers an interesting opportunity to investigate both journal ranking and PAR deals, both of which are united in Norwegian science policy. Norway has a national reimbursement program for publicly funded research institutions, whereby funds are allocated to participating institutions according to the number of published scientific works as well as their quality. To decide on the quality, the arrangement applies a simple two-level journal ranking, where prequalified journals are ranked according to their quality and importance (HK-dir, n.d.). The journal ranking system enables investigations of how choices of gold and hybrid open access journals are distributed across the levels over time since all publicly funded publications with at least one author affiliated with a Norwegian institution are registered in a national database. Norway has also implemented PAR deals, which came into effect in 2019. This was a proposal initiated in the *National goals and guidelines for open access to research articles* (Ministry of Education Research, 2017) and later strengthened by the endorsement of Plan S (cOAlition, 2018).

The motivation for the study lies in expanding the knowledge of the reward system of science and its effect on open access, which in turn connects to current science policy and its preference for immediate open access and PAR deals. These cause both tension and relief with respect to researchers' choices of journals, particularly in the case of hybrid open access journals, which in most policies are only deemed eligible if part of a PAR agreement. Consequently, there is a need to recognise and understand how policy measures interact with researchers' preferences. To extend the findings beyond the Norwegian context, this study also investigates the relationship between the Norwegian two-level journal ranking and other established journal metrics.

The research objective is operationalized in the following research questions, which guide the study together with a review of relevant literature:

RQ1: What is the effect of central handling (publish-and-read deals) on researchers' choices of hybrid open access publishing?
RQ2: What is the effect of journal ranking (“normal” and “high” level) on the distribution of gold open access and hybrid open access articles?
RQ3: How does the varying potential for relevant high ranking gold open access journals relate to immediate open access publishing within the different research areas and disciplines?

## Review of relevant literature

The review is organised in three subsections, starting with outlining useful theoretical perspectives on the reward system in science that help to explain the different incentivizing roles of gold and hybrid open access journals. This is followed by a brief introduction to open access, article processing charges (APCs), and the introduction of PAR deals. Finally, after accounting for the Norwegian context, the review is used to highlight expectations for the descriptive analysis to follow.

### The reward system of science and the role of journals

In the late 1950s, Merton conceptualised the reward system of science and claimed that the “institution of science has developed an elaborate system for allocating rewards to those who variously live up to its norms” (1957, p. 642). The four institutional norms of science are universalism, communism, disinterestedness, and organised scepticism, which together govern the main objective of science: the “extension of certified knowledge” (Merton, 1973, p. 270). Thus, a researcher is rewarded for contributing to the scientific knowledge base, a reward that comes in the form of symbolic and material recognition by peers. According to Merton, this recognition constitutes the main motivation for researchers to engage in science and forms the basis for career advancement in a meritocratic system. The contribution must be published in order to be made public, publication is thus the core element in the reward system of science (Desrochers et al., 2018), and is considered to be the currency for academic careers (Elliott, 2013).

The journal has become important for the accumulation of reputational capital, up to the point that the journal in which a researcher's work is published can be perceived as more important than the actual research itself (Steele et al., 2006; Macdonald and Kam, 2007; Heckman and Moktan, 2020). Thus, a common strategy among researchers is to submit articles to the most prestigious journal in the relevant field that might publish them (Nosek and Bar-Anan, 2012). Publishing in reputable journals of prestige is a powerful incentive that researchers will almost always take into consideration (Rushforth and de Rijcke, 2015) and has its counterpart in commercial strategies where publishers position their journal brands in the continuous quest for recognition in science (Khelfaoui and Gingras, 2021).

### The reward system and the role of metrics

Merton holds that the shaping of the reward system is a result of evolution over centuries, one that is probably far from finished (Merton, 1957). The evolution of the reward system has turned to quantitative research evaluations either at the expense of, or in addition to, traditional peer review, with the result that the symbolic capital of researchers has become both more visible and summarised (Desrochers et al., 2018). The science system is nowadays flooded by a wave of scientometrics indicators, which are easily developed and simple to use in research assessment, along with existing and disputed ones (Cronin, 2014). This development has been called “the metric tide” (Wilsdon et al., 2015).

The central role of journals has spawned journal ranking as one type of scientometric indicator. While there are many implementations, the journal impact factor (JIF or just “impact factor”) is the most controversial metric precisely because it is applied in the assessment of the individual article or its author(s). Impact factors, therefore, have a significant influence on choices of where to submit articles (Huang et al., 2019; Gaston et al., 2020). JIF was originally developed for library acquisition strategies (Garfield, 1972), and is calculated based on the two-year rolling average number of citations in a journal and amounts to a single numeric value (Garfield, 2006). While easy to use and understand, the use of impact factor in research assessment is considered bad practise as there is no link between an article's “quality” (measured by the number of citations) and the impact factor of its journal. There is a high degree of skewness in the distribution of citations within a journal, whereby few articles account for most of the citations (Antonoyiannakis, 2019), therefore, impact factors are inappropriate as a proxy for quality on the article level (Seglen, 1992, 1997). It has been claimed that the discouragement of using impact factors can be extended to all journal rank systems (Brembs et al., 2013).

The above arguments have not prevented the use of journal ranks in formal review processes: 40% of research-intensive universities in North America apply impact factors in processes relating to the review, promotion, and tenure (McKiernan et al., 2019). Similarly, in Europe, 75% of 186 universities in a European University Association survey report, applied impact factor to evaluate researchers' output (Saenen et al., 2019). Publishing in high ranking journals has led to the introduction of monetary rewards (Quan et al., 2017), which means that journals literally can be regarded as “mints,” with the impact factor being a journal's actual value (Biagioli and Lippman, 2020).

While scientometric indicators allow for insights into reward dynamics, they also have the potential to transform behaviour with incentives in accordance with Goodhart's law; once a measure itself becomes the goal, it ceases to function as a measure (e.g., Paul-Hus et al., 2017). A study by Rushforth and de Rijcke (2015, p. 136) found evidence that described the ‘field of biomedicine as captured by the JIF' and journal ranks thus shape knowledge production (Ràfols et al., 2012). This development has spawned calls for more responsible use of scientometric indicators (Hicks et al., 2015; Wilsdon et al., 2015).

### Gold and hybrid open access

The formal start of open access took place through a series of declarations in 2002 and 2003, with the Budapest Open Access Initiative in 2002 as the first and most widely cited (Chan et al., 2002). The vision was the “free and unrestricted online availability” of the scientific literature, enabled by the Internet. Green open access presupposes publication in subscription-based journals, while gold and hybrid open access requires that expenses for publication must be covered, for example, by article processing charges (APC). While APCs are always present in hybrid journals, the majority of gold open access journals do not depend on APCs, such journals are often called diamond open access journals (Bosman et al., 2021).

Hybrid open access was from the start meant as a risk-free mechanism for publishers to convert subscription-based journals to open access and designed to be attractive for journal owners that were reluctant to convert to open access (Prosser, 2003). Several challenges are associated with hybrid open access, most of an economic nature. APCs are significantly higher in hybrid journals (Laakso et al., 2016; Pinfield et al., 2016, 2017), one extreme example is the top-tier journal *Nature*, which announced charges of € 9,500 to make a single article hybrid open access (Brainard, 2020).

A serious allegation is ‘double dipping, which is when hybrid journals receive revenue through subscription fees, even for open access articles for which they have previously received payment (Shieber, 2009). Evidence shows that this practise ranges from partial double dipping to full double dipping, even when journals have a “no double-dipping” policy (Mittermaier, 2015). Double dipping continues to be an obstacle that must be avoided for a successful transition to open access (Björk and Solomon, 2014), and this also serves as a motivation for PAR deals. Today, the hybrid option is offered by the vast majority of subscription-based journals from major publishers (Björk, 2017).

Article processing charges are important for researchers' choice of journals. Surveys show that two of the most important reasons for not choosing immediate open access publication are that researchers are either not willing to pay for it themselves or not able to obtain funding to pay for it (Nature Publishing Group, 2015). In a survey with 2,112 respondents at universities in North America, Tenopir et al. found that willingness to pay APCs relate to access to research funds. There was more willingness to pay APCs and higher APCs if the costs were centrally handled by a university library or through grants, than if they had to be paid from either individual funds or project funds, and more than half of the respondents were not willing to pay anything at all (Tenopir et al., 2017). Such reluctance to pay APCs is partly compensated by funding mechanisms: Solomon and Björk found that research grants and institutional funds were the main financing mechanism for gold open access journals with higher APCs, while personal project funds played a bigger role when APCs were in the lower APC price brackets (Solomon and Björk, 2012).

### Authors' perceptions of gold open access journals

In a meta-synthesis on attitudes towards open access, Togia and Korobli conclude that quality and reputation are the most important factors when choosing a journal, and these factors are prioritised over gold open access options. The gold model brings concerns over the “author pays” model, the quality of peer review, and the impact of the journals (Togia and Korobili, 2014). In addition come concerns over predatory journals (Butler, 2013; Zhao, 2014), which often are associated with gold open access by their exploitation of the “author pays” business model. Predatory journals pose serious threats, not only to researchers but also to the academic knowledge base. Articles from predatory journals are included in indexes and pollute the scientific record (Moher et al., 2017; Severin and Low, 2019).

Authors favour legacy journals hosting green and hybrid open access, which generally have acquired a stronger meritorious role in the reward system than their gold open access counterparts (Anderson, 2004; Björk, 2013; Solomon et al., 2013). University faculties are often conservative in their acceptance of open access (Tenopir et al., 2017) and aspiring researchers may feel obliged to choose journals that their faculty ranks as being of high quality (Nicholas et al., 2017, 2020; Dalton et al., 2020; Heckman and Moktan, 2020). These effects may have reinforcing outcomes on the coverage of open-access journals in a discipline. In their study of the business fields, Laakso and Björk did not find any top-tier gold open access journals within the discipline. The authors suggest this is a side-effect of evaluation practises encouraging submissions based on journal ranking lists, making it difficult for new journals (open access or not) to gain a foothold (Laakso and Björk, 2021).

Negative attitudes towards gold open access journals are not uncontestably supported by studies that have investigated journal metrics. Two studies found that APC-based open access journals were approaching the same citation impact as subscription-based journals, while non-APC-based journals were lagging behind (Björk and Solomon, 2012; Solomon et al., 2013). A different study found that gold open access journals had statistically significant lower average values in three journal metrics in 26 of 27 research areas in Scopus (Erfanmanesh, 2017). Huang et al. found that open access had a positive effect on the JIF scores for medical journals (Huang et al., 2019).

### Publish-and-read deals

Historically, open access policies have viewed gold and green as equal pathways to open access (Crowfoot, 2017), while hybrid has been discouraged in policies because of concerns of economic “double-dipping” and high APCs. Thus, the hybrid has been growing slowly, due to financers and institutional funds usually have declined to support the hybrid financially (Kita et al., 2016). A recent study of Elsevier's journals found a moderate uptake of hybrid open access, with 3.7% of all articles being published in hybrid open access journals in 2019 (Jahn et al., 2021), a share coherent with large-scale studies that have shown a similar and modest uptake (Piwowar et al., 2018). Nevertheless, hybrid open access has acquired an important strategic role in Plan S due to the explicit goal of converting subscription-based journals to gold open access (cOAlition, 2018) and is thus given the role as the transformative element that it originally was designed for (Prosser, 2003). Its practical implementation is through PAR deals, which are agreements between publishers and consortia of research institutions that regulate both reading and publishing rights while removing the risk of double dipping. An important aspect of PAR deals for this study is that they bring considerable relief to researchers at participating institutions that want to publish open access in legacy journals but are unwilling or unable to pay APCs. Any reluctance to pay for publishing likely disappear with PAR deals since the cost of publishing is handled centrally by funders or universities (Togia and Korobili, 2014).

The journal's role in the reward system and journal ranks is also recognised as important in the context of PAR deals. A report commissioned by the European University Association (EUA) on PAR deals and the future of scholarly publishing, emphasises that a barrier to open access publishing is the need to publish in high impact journals for career advancement (van Barneveld-Biesma et al., 2020). As a relatively new phenomenon, the effect of PAR deals has not been explored empirically in the literature, there is however uncertainty whether PAR deals will be the transformative deal breaker they are intended to be (Borrego et al., 2020).

### Open access and disciplinary differences

Disciplinary differences in open access publishing have been investigated in a review by Severin et al. (2020) who synthesised 11 bibliometric studies of open access prevalence and publishing patterns across academic disciplines over time and found large disciplinary differences subsumed under five research areas. The most relevant findings for the present study were that within the medical sciences the level of open access is dominated by gold open access journal publishing, with other modalities being less relevant. Researchers in the medical sciences can choose from many high-quality gold open access journals, and generally have sufficient funding available. Journal reputation and impact factors are considered more important than open access when researchers choose journals to submit (ibid.).

The natural and technical sciences have the highest prevalence of open access among all research areas, driven by a culture of preprints and green open access. Research is mainly funded by project-specific grants, making it possible to fund APCs for gold or hybrid journals (Severin et al., 2020). In the social sciences, monographs are important to work products in addition to articles, with lower levels of open access than in the medical and natural and technical sciences. Journal prestige and journals with a high impact factor are important signs of recognition as also found in Hessels et al. (2019). Levels are mostly driven by green open access, with few existing gold open access journals in specific subdisciplines that are considered of less importance and with limited impact and readership. Furthermore, social scientists have reported significant difficulties in securing grants and funding for APCs, as most of their research is not connected to project-specific funding (Severin et al., 2020). The humanities have the lowest levels of open access, and also have monographs as important work products (ibid.). Journal ranks and impact factors play a lesser role than an informal journal hierarchy, although choices of journals operate within a symbolic economy of prestige. There is little information on the different open access modalities in the humanities although there are indications that green dominate and hybrid open access publication is of central importance. Most research in the humanities does not receive project-specific funding, making it difficult to handle APCs (Severin et al., 2020).

### Context and policy background of the research topic

In 2006, Norway introduced a reimbursement program for publicly funded institutions, where funds are allocated to an institution based on the number of articles (or author-shares in case of co-authoring) their researchers have contributed, as well as where the articles are published (HK-dir, n.d.). Articles will only be considered for reimbursement if published in a journal registered in a prequalified list, where journals are assigned on either level one (“normal”) or two (“high”). This assignment is carried out according to a journal's perceived rank and prestige, articles published in a journal on level high also yield a higher monetary return to the institution. The argument for assigning a journal to the highest level is that “the international impact of the channel is of utmost importance and further that ‘in many academic fields, this is measured by the impact factor^1^” (HK–dir, n.d.). Thus, impact factors are directly taken into consideration by the expert panels nominating journals for the most prestigious level, while any open access options a journal might offer are not taken into consideration. The use of the two-tier journal ranking system is designed ‘to create an incentive for researchers to have their work published in the most prestigious channels within their field of study' (HK–dir, n.d.) and is partly a measure to prevent effects found in Australia, where a national indicator spawned an increase in production rates, albeit mainly in lower ranked journals (Butler, 2005; Schneider, 2009). In a given year, and within each discipline, the journal rank limits the number of articles on the high level to *c*.20% and consequently *c*.80% of articles on the normal level. This is not to be confused with that *c*.20% of the *journals* within a discipline are placed on level high. The model has since been implemented in Denmark and Finland and has inspired changes in similar arrangements in Flanders, Belgium, and Poland (Sivertsen, 2018).

With the launch of the *National goals and guidelines for open access to research articles* in 2017 (Ministry of Education Research, 2017), open access was tied to the reimbursement program by making depositing of all research articles mandatory (regardless of open access status). At a later unspecified time, only deposited articles would be eligible for reimbursement^2^. The guidelines, together with Plan S and global initiatives such as OA2020 (Max Planck Institutes, n.d.) advanced PAR deals and thus addressed the key policy question about the economic concerns with hybrid publishing (Björk, 2012; Laakso et al., 2016). As of 2021, Norway has PAR arrangements with 10 publishers^3^, of which six were effective from 2020, while two deals covered parts of 2019. Hybrid open access publishing outside PAR deals is not reimbursed by any Norwegian institutional open access fund, which is reserved for gold open access publishing. PAR deals also to some extent support gold open access publishing by including gold journals in the portfolio of journals offered to researchers, PAR deals thus function similarly as dedicated institutional funds.

The main concept this study brings into the methodology section is that academic journals occupy a prominent position in the reward system of science. Consequently, journal ranks have become an influential part of researchers' choice of outlet. Gold open access journals are generally perceived as less prestigious than their legacy hybrid counterparts, but they have the advantage of being lower priced and supported by institutional funds. By contrast, hybrid open access has been discouraged in policies and was not supported financially until the introduction of PAR deals, which also eased researchers' concerns about paying APCs. The different research areas have different open access publishing cultures that depend on gold open access journal availability, their status, and access to funds.

The above-presented review and context suggest several expectations for the coming analysis. Initially, there is an expectation of similar levels in both gold and hybrid open access, as found in the literature (Martín-Martín et al., 2018; Piwowar et al., 2018), with higher overall levels and growth of gold open access, and lower levels and slower growth of hybrid open access up until the introduction of PAR deals. Further, there is an expectation of disciplinary differences in open access uptake similar to what has been reported in previous studies (Severin et al., 2020).

- In the case of RQ1, there is an expectation of strong growth in choices of hybrid journals due to the “easiness” of PAR deals. Although not supported by any studies in the literature, there is reason to believe that at least some of the non-PAR hybrid is replaced by PAR-hybrid due to the attractiveness of central handling of APCs.
- In the case of RQ2, there is an expectation that gold open access journals on the high level are chosen less often than gold journals on the normal level, due to a lack of prestigious gold open access journals. In the case of hybrid, I have not been able to find studies that suggest that journal ranking would increase or decrease researchers' propensity to choose hybrid open access, once a journal with the hybrid option is chosen. In addition, since the distribution of hybrid choices between the normal and high level is unknown, there are no grounds for expecting that the distribution will change with the introduction of PAR deals.
- In the case of RQ3, the gold uptake on the high level is expected to be a function of the availability of relevant journals within the disciplines in each of the main research areas. However, there are no clear expectations of a similar relationship to the gold uptake on the normal level or that publishing on the high level is driven into hybrid journals.

## Methods and data

Before addressing the research questions, the first subsection will give a synopsis of the preparation of data, assumptions, and decisions relevant to the research questions, together with a presentation of the overall development of open access in Norway.

### Datasets: Enhancing data with open access status

The central source of data for the study was Cristin (Current Research Information System in Norway), a database that covers the production of 102 institutions in the health, institute, and higher education sector. Cristin holds a near complete record of Norwegian publicly funded research and is a type of bibliographical database that holds great potential for monitoring open access and policy compliance (Pölönen et al., 2020). Cristin is also used for depositing full texts of published articles to institutional repositories since all member institutions of Cristin have their repositories connected to the system.

The second main source of data was the list of all eligible journals where all articles were published. This is a list maintained by HK–dir (n.d.). Both sources were enhanced by the following procedures:

#### Articles

The database was initially limited to a subset of original scientific journal articles resulting in a dataset of 186,621 journal articles in the period 2013–2021. The dataset was retrieved on 28 February 2022, a date selected because registration of articles due for reporting for 2021 was adjourned at most of the 102 Cristin-institutions.

Quantitative studies of open access at the country level face two main challenges (Pölönen et al., 2020). The first is the lack of records in traditional bibliographical databases (Huang et al., 2020a), the most popular being the proprietary data sources Web of Science (WoS) and Scopus. This challenge is largely avoided by using Cristin, due to the near completeness of its data.

The second challenge is to identify the open access status of each article, particularly hybrid, which has been difficult to identify due to a lack of information in databases and a uniform way of classification (Laakso and Björk, 2016; Björk, 2017). This is a remedy by Unpaywall, which has been called a gamechanger in monitoring open access uptake (Else, 2018; Robinson-Garcia et al., 2020). Besides Unpaywall, the Directory of Open Access Journals (DOAJ), which holds information on gold open access journals was used, in addition to reports from publishers on the hybrid in PAR deals.

Each article was assigned a single open access status by one of the above sources, one of either gold, hybrid, green, deposited, or closed. Unpaywall's own open access classification was taken “as is,” except for “bronze,” which was reclassified as “closed” due to its lack of compatibility with any common definition of open access. Of the 186,621 articles, 184,396 contained some kind of information about their open access status, the remaining were classified as “closed.”

#### Journals

The list of publication channels in the *Norwegian Register for Scientific Journals, Series, and Publishers* was downloaded on 15 February 2022. The list included 33,768 journals assigned to one of 87 disciplines within four research areas (humanities, social sciences, health sciences, and natural sciences and engineering) according to the disciplinary hierarchy in the Norwegian publication indicator (HK-dir, n.d.). Of those, 28,319 were on the normal or high level in 2021. The journal list also holds information on the journal's placement on level normal or high throughout the period. Each journal record was enhanced with data from DOAJ, the Scopus journal list, and Scopus CiteScore data by joining records on one of the possible combinations of ISSN values in the sources. Scopus CiteScore journal list contains metrics data on CiteScore, Source Normalised Impact per Paper (SNIP), and Scimago Journal Rank (SJR) (Scopus, n.d.).

CiteScore, a similar metric as the impact factor, is calculated by the number of citations to publications in a journal in the previous 3 years, divided by the number of published items in the journal. The number of items also includes letters, editorials, and other types of material.

SNIP is a field normalised calculation of journal impact based on the ratio of a journal's average number of citations and the “citation potential” in the field given by a wider reference set of comparable journals. The citation potential is the number of citations that a journal could be expected to receive for its subject field, e.g., the average of the comparable journals in the reference set.

Scimago Journal Rank is also a normalised measure of journals and is computed by network analysis of citations received by the journals. The methodology includes both the number of citations and their source, with citations from journals with higher levels of prestige having a higher value than those from journals with lower prestige.

All journal and article data were imported, processed, and transformed into a tidy format in R, which also was used to generate plots and figures presented in the results section. The dataset used in the study is made available at https://doi.org/10.18710/TBXXCC.

Table 1 presents an overview of the data and the key numbers of the most important variables used in the study.

**Table 1:** Overview of data, sources, and enhancements.

	**No. records**	**Key data/numbers**	**Retrieved (day, month, year)**
**Articles** (article records 2013–2021)	186,621	Has DOI : 169,450 Has ISSN or EISSN: All Has uploaded file(s): 87,104 Has link to a Norwegian repository: 66,745 Returned any open access status: 184,396	28.02.2022
+ Unpaywall	All containing DOI	Returned any value: 166 383	28.02.2022
+ PAR publication reports	14,336	Within PAR deals: 9006	28.02.2022
**Journals** (journal records)	33,768	Level one (“normal”) or two (“high”) assignment (2021): 28,319	15.02.2022
+ DOAJ	17,428	Open access information merged in: 5,026	15.02.2022
+ Scopus journals	42,474	Information merged in: 21,957	15.02.2022
+ Scopus CiteScore	25,990	Metrics merged: 18,192 (2020 metrics)	15.02.2022

### Overview of statistics relevant for the research questions

**Figure 1:**
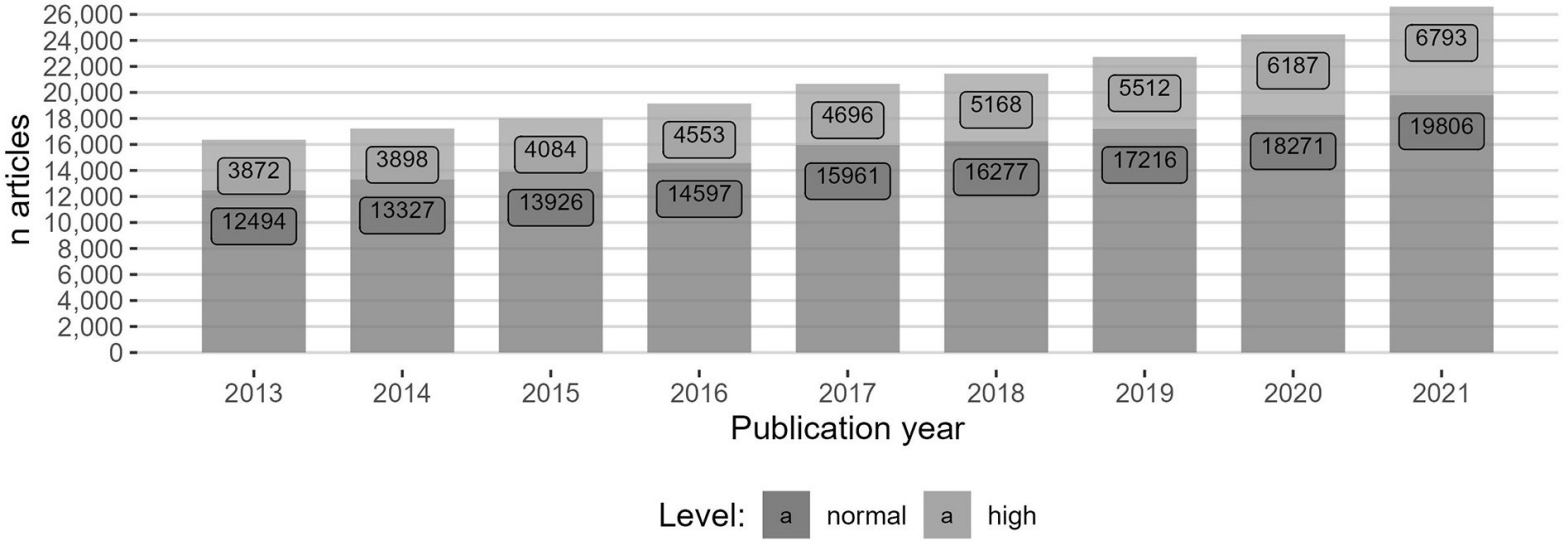
Absolute growth in Norwegian journal articles in the period 2013–2021, including their distribution between level normal and level high according to the Norwegian journal ranking.

Figure 1 gives an overview of the growth of articles in the period and the distribution of articles at the normal and high levels. The number of articles increased from 16,366 in 2013 to 26,599 in 2021 with an annual average growth rate of 5.9%. The share of articles on the high level in the period varied between 22.6 and 25.5%.

**Figure 2:**
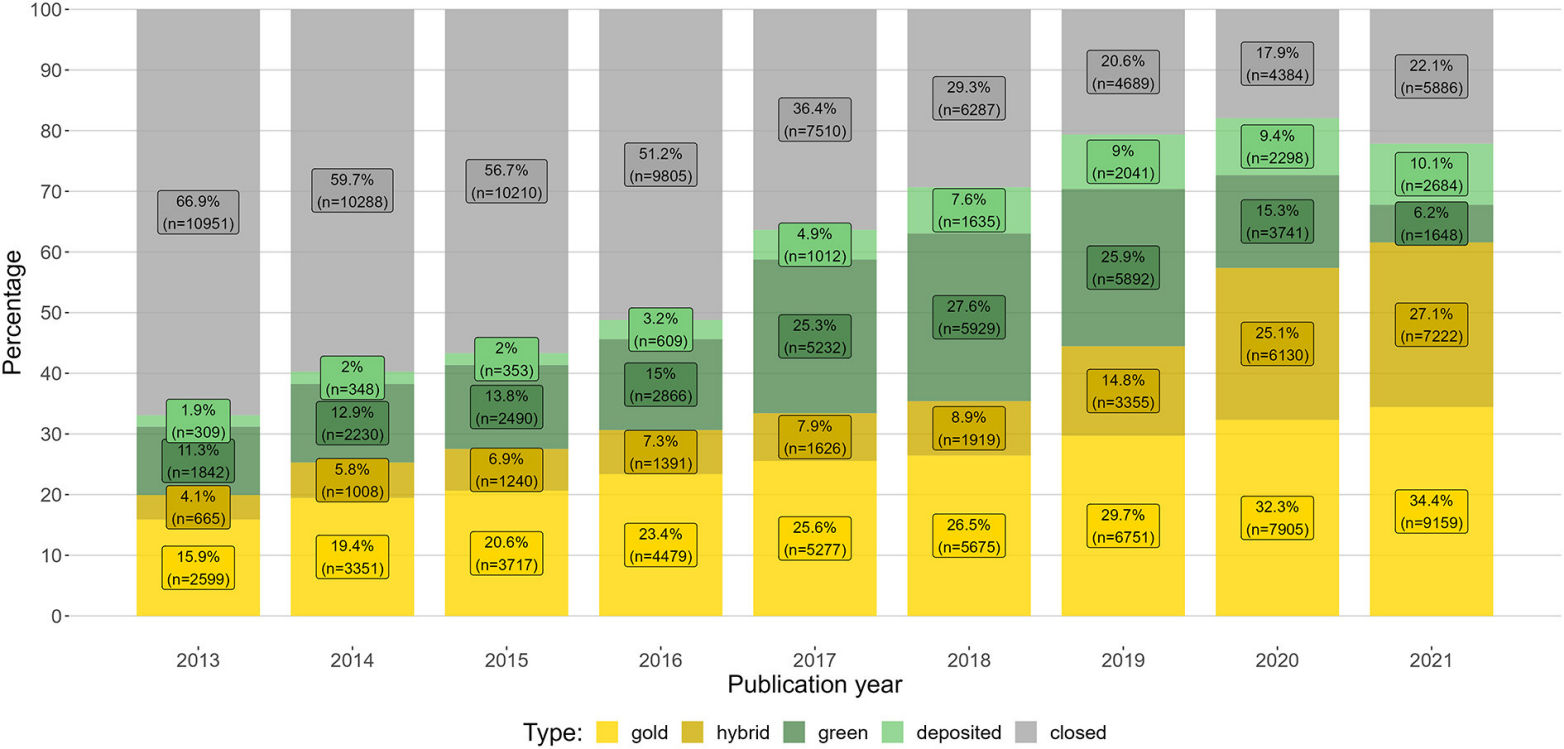
The distribution of Norwegian journal articles' open access status in the period 2013–2021, with shares and numbers of gold, hybrid, green, deposited, and closed articles.

Figure 2 presents the development of open access articles in the period 2013–2021, with shares and numbers of gold, hybrid, and green open access, as well as deposited (but not yet green and openly available) articles (light green) and closed articles.

The overall level of articles with open access increased from 31.2% in 2013 to 67.8% in 2021 (not including deposited articles), and the annual average growth rate was 4.6%. Up until the introduction of PAR deals in 2019, gold open access had a three to four times larger share of output than hybrid open access, while hybrid had strong growth in the period 2019–2021 following the PAR deals.

It should be noted that Figure 2 is a snapshot taken at a given moment. While hybrid and gold open access articles acquire their open access status at the time of publishing, green open access is in principle obtainable at any time after publication. In addition, deposited articles (shown in light green in Figure 2) do not always reach the status of green open access. Even though presumably most embargo periods have expired, the share of deposited articles after 2017 is quite large, pointing to an unrealized potential for green open access even in the case articles are deposited. The potential of the 10.1% deposited access articles in 2021 can therefore be expected to only be partially realised. This effect is likely connected to the introduction of mandatory depositing in the National Guidelines in 2017.

When considering total output, there are large differences between the four research areas. In 2021 the humanities held a 6.4% share of total articles, the social sciences held a share of 18%, health sciences held a share of 30.7%, and natural sciences and engineering held a share of 44.9%.

**Figure 3:**
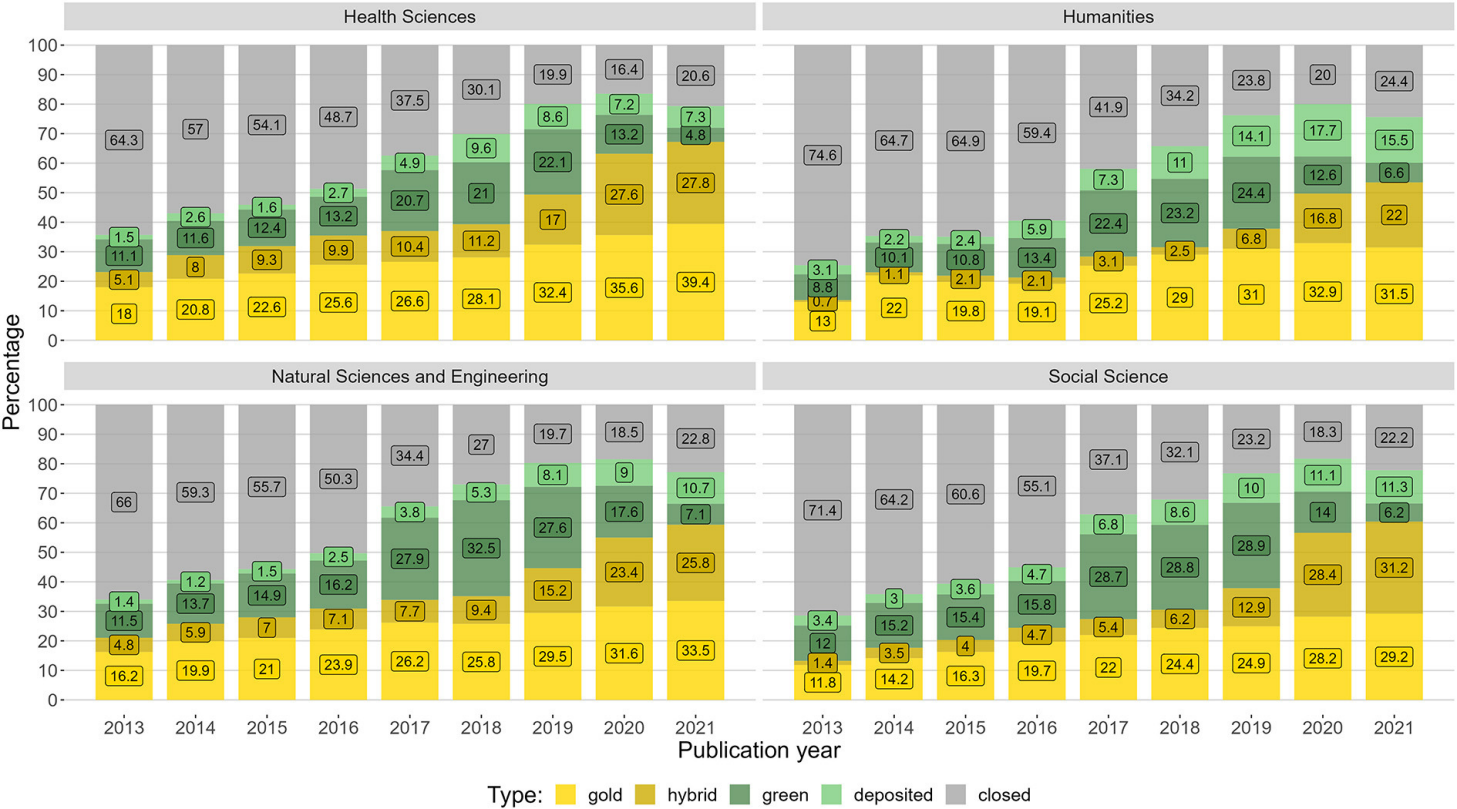
The distribution of Norwegian journal articles' open access status in the period 2013–2021, with shares of gold, hybrid, green, deposited, and closed articles. The plot is separated on the four main research areas.

Figure 3 presents the development of open access broken down in the four research areas, reported in percentages of their respective shares.

Even though the levels are consistent with what has previously been found (Severin et al., 2020), the four research areas are relatively similar with respect to the shares of the different types of open access. The health sciences, natural sciences, and engineering have the highest levels of gold and hybrid, while the humanities also have high levels of gold. The humanities and social sciences have low levels of hybrid up until the introduction of PAR deals, and also have the lowest overall level of open access in the period.

As a final preparation before embarking on the research questions, the alignment between the Norwegian journal ranking system and other systems of journal indicators was investigated. This was performed to be able to extend the findings in the study outside the Norwegian context.

**Figure 4:**
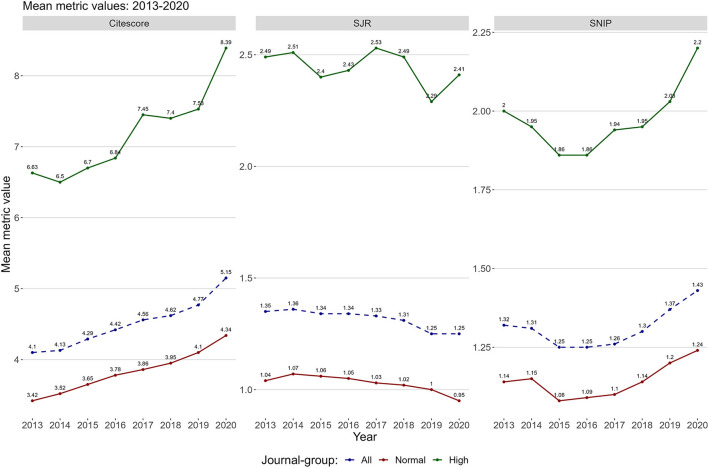
The yearly mean SJR, SNIP, and CiteScore value in the period 2013–2020 for journals with at least one publication in the period 2013–2020 (*n* = 16,056). The journals are grouped by their assignment on the normal level (red line) or on the high level (green line). The dashed blue line represents the mean value for all journals irrespective of level assignment.

Figure 4 shows the relationship between the Norwegian system and three journal metrics (SJR, SNIP, and CiteScore) in the period 2013–2020. The comparison was performed on all journals with at least one publication with a Norwegian affiliation in the period (*n* = 16,056). The mean number of yearly journals with publications in the selection was 6,292 in the period (ranging between 5,390 and 7,067). The coverage of journals with a metric value in the period was respectively mean 81.0% for SJR in the period (ranging between 79.7 and 83.4%), mean 81.0% for SNIP in the period (ranging between 79.3 and 83.4%), and 81.6% for CiteScore in the period (ranging between 80.3 and 83.4%). The yearly mean metric values were calculated respectively for the set of journals assigned to each level high and normal.

Figure 4 shows the mean value for each metric for all journals placed on level high (green line), which are consistently higher than the mean value of all journals placed on level normal (red line). For reference, the mean value of all journals regardless of level assignment is presented by the dashed line. There is a high degree of alignment between all three journal metrics and the Norwegian system. Consequently, the Norwegian journal rank can be viewed as a stratified metric system in alignment with other widely used metrics. This is also consistent with findings in a similar investigation in Finland, which has a similar system to Norway (Saarela and Kärkkäinen, 2020).

In summary, the initial findings show strong growth in both the number of published articles and the overall levels of open access in the period. The alignment between the two-tier-system and established journal ranking was persistent over time, it can thus be argued that findings from this study can be extended to other systems where journal ranks are incorporated.

## Results

### RQ1: What is the effect of central handling (publish-and-read deals) on researchers' choices of hybrid open access journals?

The effect of PAR deals was found by isolating the levels of hybrid open access inside and outside PAR deals. The levels are presented in percentages and can be considered to be “market shares in a growing market,” since the total number of publications increased during the period (Figure 1).

**Figure 5:**
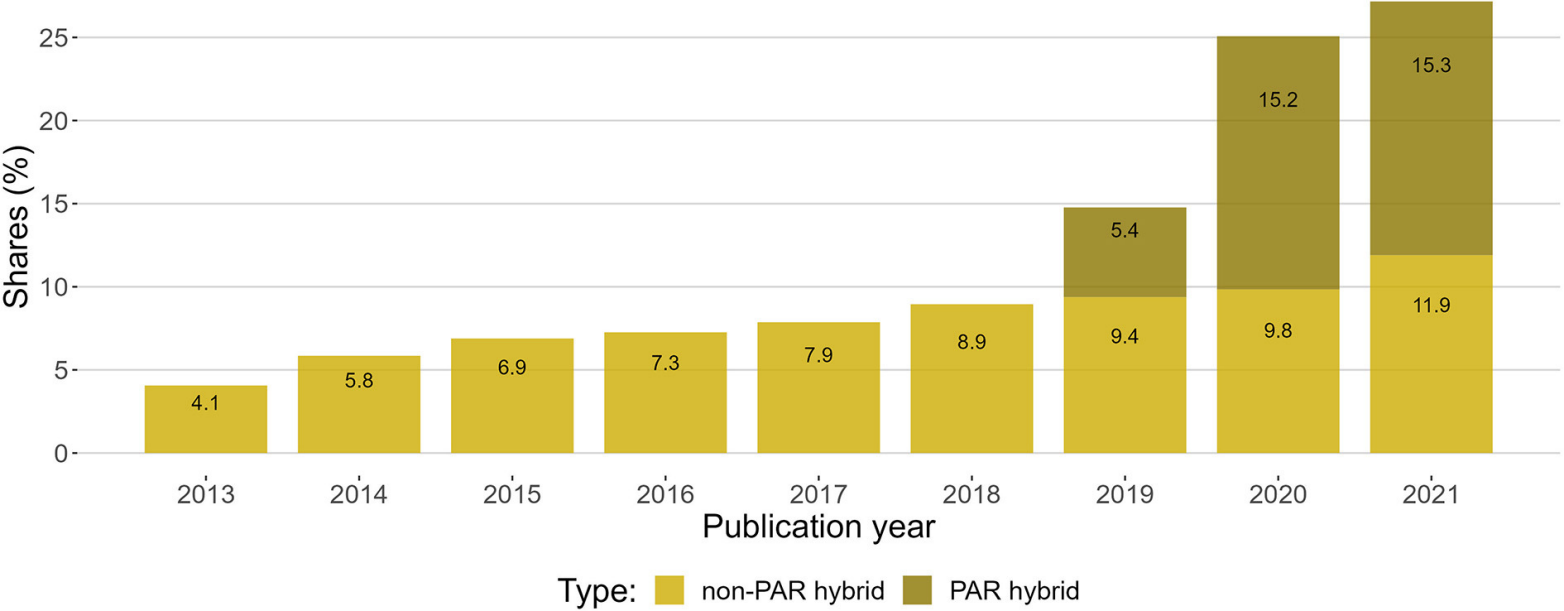
Shares of PAR hybrid and non-PAR hybrid open access articles of total output of articles in the period 2013–2021.

Figure 5 shows the growth in hybrid open access articles and the overall effect of PAR deals.

The increase in the growth rate of non-PAR hybrid and total hybrid is presented in Table 2.

**Table 2:** Annual growth rate of hybrid open access articles calculated from the previous year, starting in 2013.

	**2014**	**2015**	**2016**	**2017**	**2018**	**2019**	**2020**	**2021**	**Average annual growth rate**
Hybrid excl. PAR	1.8	1.0	0.4	0.6	1.1	0.4	0.5	2.0	1.0%
Hybrid inc. PAR	1.8	1.0	0.4	0.6	1.1	5.8	10.3	2.1	2.9%

The publish-and-read deals increased the overall levels of hybrid open access from 8.9% in 2018 to 27.2% in 2021. When considering the overall levels of open access (Figure 2), the increase in hybrid articles translates to more immediate open access, but it is not clear that the increase also translates to more open access overall. The growth in hybrid articles corresponds with a similar decrease in green open access articles, which is best seen in the data for 2020 and 2021 in Figure 2.

Norwegian PAR-deal hybrid open access has primarily added to, rather than replaced non-PAR hybrid open access. To cheque if the level of non-PAR hybrid in the period 2019–2021 was unaffected by Norwegian PAR deals, the hybrid production of all Norwegian universities was investigated, comparing whether hybrid articles were a part of PAR deals or not.

**Figure 6:**
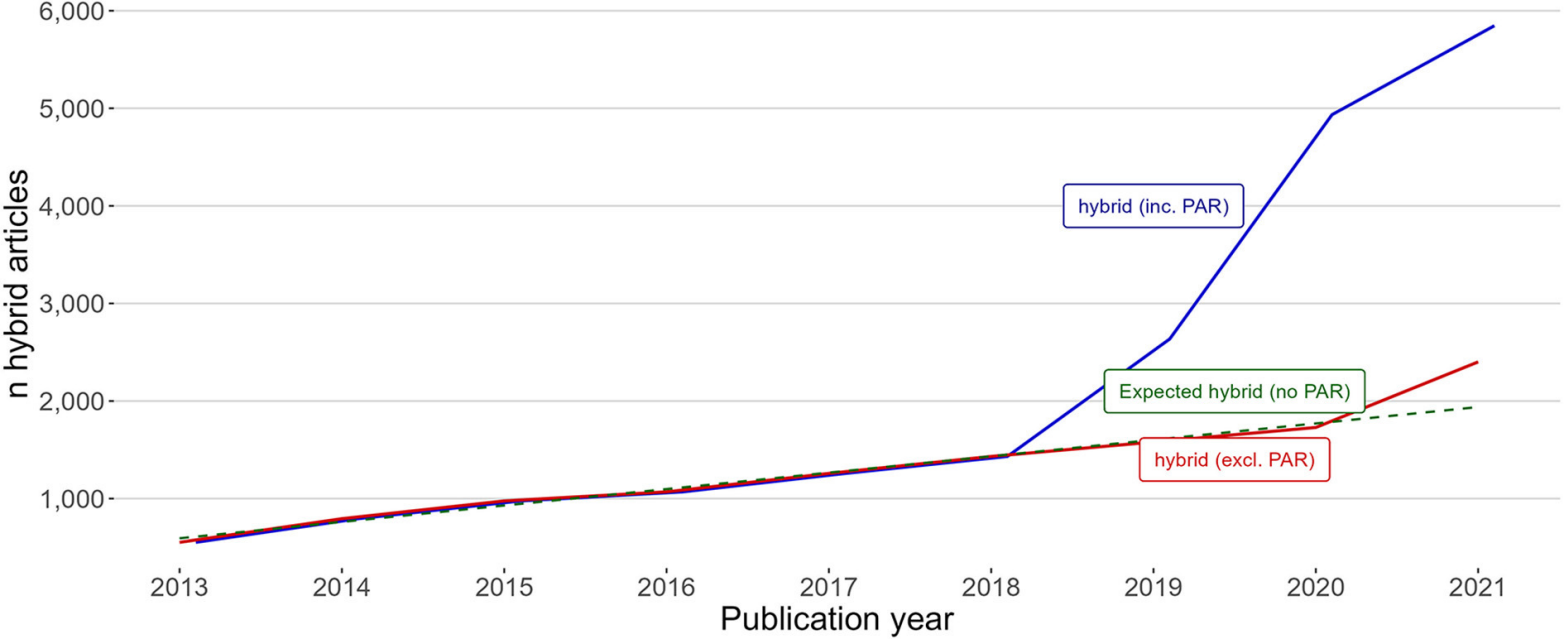
All 10 Norwegian universities' production of hybrid open access articles in the period 2013–2021, separated by hybrid inc. PAR hybrid (blue line) and hybrid excl. PAR hybrid (red line). The dashed green regression line indicates expected level of hybrid open access publications without the introduction of PAR deals. The prediction is based on 2013–2018 data.

Figure 6 shows this difference for all 10 Norwegian universities, these institutions also accounted for the majority of hybrid open access articles in the PAR deals.

The blue and red lines in Figure 6 are in full alignment until the introduction of PAR deals in 2019. Without PAR deals, my expectation was that the red line would be in close alignment with the dashed regression line, which is a linear prediction of hybrid uptake for 2019–2021 based on the production of hybrid open access articles in the preceding years. However, non-PAR hybrid open access articles increased in 2021, indicating that PAR hybrid came in addition to non-PAR hybrid.

**Figure 7:**
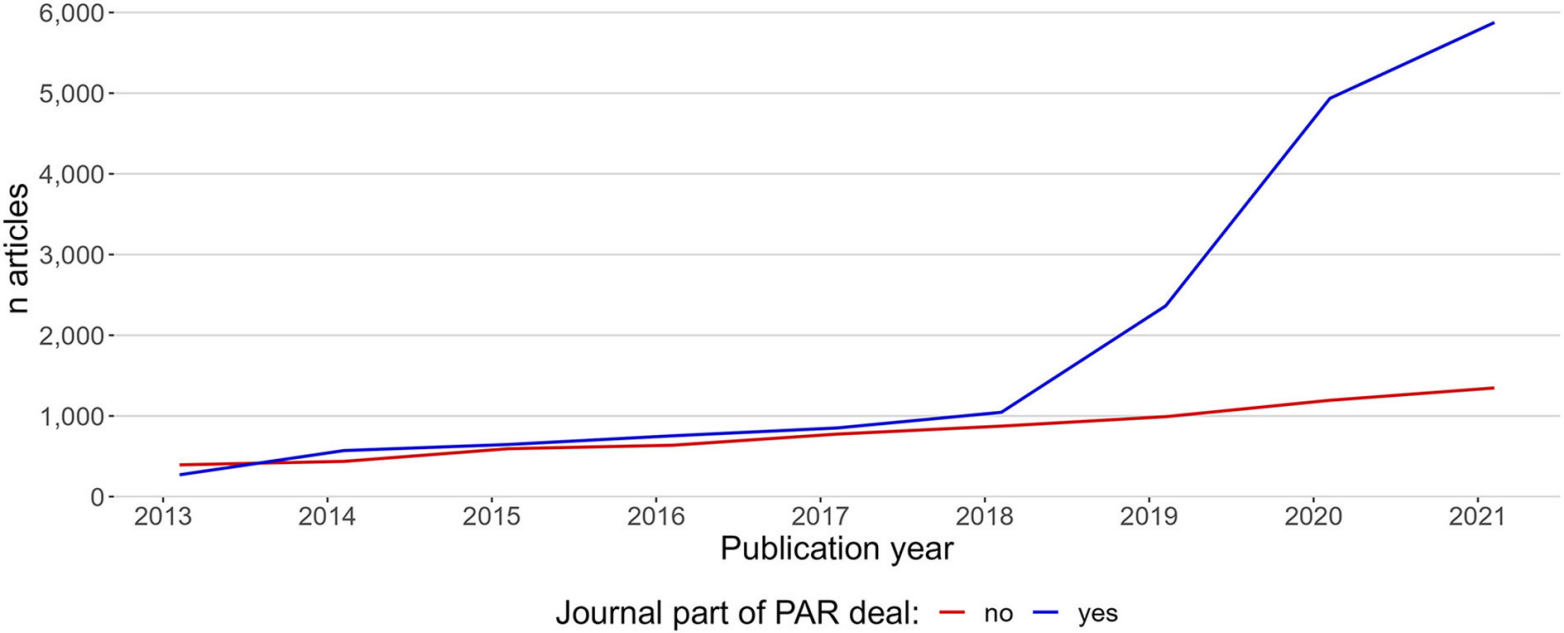
The total production of hybrid open access articles in the period 2013–2021, separated by whether articles were published in journals that are a part of PAR deals (blue line) or not part of PAR deals (red line).

The same effect was found when viewing the hybrid production rate from the perspective of the journals. Figure 7 shows the total production of hybrid articles in journals with at least one publication, separated into two groups by whether the journal was part of a PAR deal or not in the period.

As with Figures 6, 7 indicates that PAR hybrid open access publications come in addition to non-PAR hybrid.

A possibility the study was not able to control for, was whether the level of non-PAR hybrid open access was affected by PAR deals in other countries. It can be noted that more than 50% of articles published later than 2017 also had international co-authors, pointing to a probable impact on the share of Norwegian non-PAR hybrid.

### RQ2: What is the effect of journal ranking (“normal” and “high” levels) on the distribution of gold open access and hybrid open access articles?

To answer RQ2, the market shares of respectively gold and hybrid open access articles were split into groups according to the journals' level at the time of publishing. This produced a 2 × 2 matrices of shares that were used to:

(a) compare the shares of gold and hybrid open access articles on the normal level.
(b) compare the shares of gold and hybrid open access articles on the high level.
(c) compare the shares of gold open access articles on the high and normal levels.
(d) compare the shares of hybrid open access articles on the high and normal levels.

**Figure 8:**
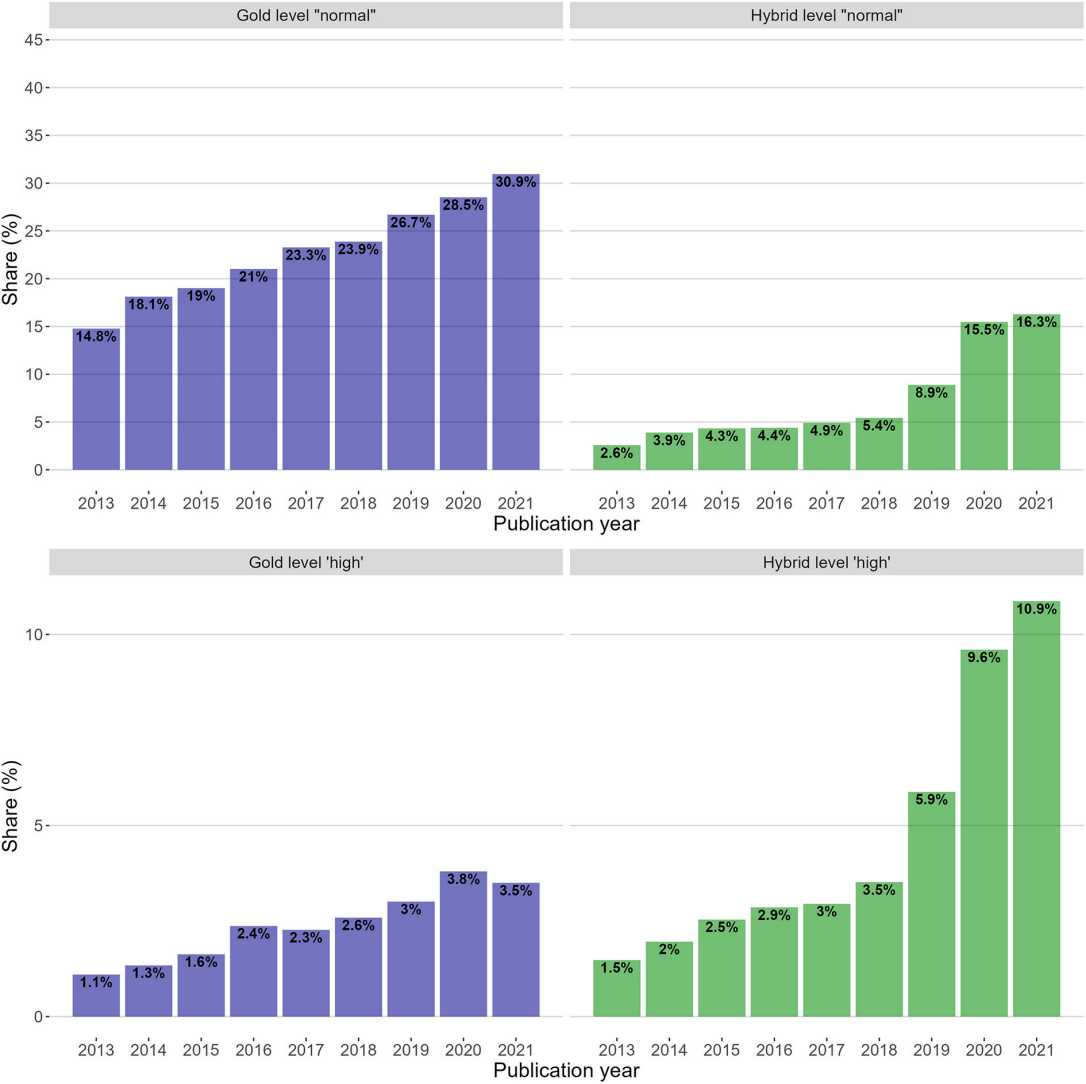
Gold and hybrid shares on the normal level (top two sub-plots) and high level (bottom two sub-plots). The scale of the *y*-axes are adjusted for comparison between levels. Each bar is annotated with the absolute share of total articles in that year.

Figure 8 aims to visualise the shares of hybrid and gold open access articles (column-wise) on each level (row wise), where each bar is annotated with the share of the total number of articles. As with RQ 1, article shares are given as percentages since the number of total publications increased during the period. The y-axes are adjusted to make the sub-plots and “market shares” more visually comparable, even though the “market” is split into two segments of different sizes. The two top sub-plots are scaled from 0 to 80% to reflect the share of publications on the normal level, whereas the two bottom sub-plots are proportionally augmented and scaled from 0 to 20%.

As previously explained, there is ~80/20 distribution of the articles per convention. While shares of articles on the same level were directly comparable, comparing shares on different levels required relative shares in accordance with the 80/20 distribution.

Table 3 presents the relative shares of hybrid and gold on their respective levels. For example, the share of hybrid on level high in 2021 was 10.9% of the total number of articles that year (as shown in Figure 8), but this accounted for 42.6% of the articles on level high. The relative shares are thus more suitable for comparing shares across levels.

**Table 3:** Relative shares of hybrid and gold on both level normal and high.

**No**.	**Open access type**	**Journal level**	**Share according to level**	**2013**	**2014**	**2015**	**2016**	**2017**	**2018**	**2019**	**2020**	**2021**
*i*	Gold	Normal	*c*.80%	19.4	23.4	24.6	27.6	30.1	31.5	35.2	38.2	41.5
*ii*	Gold	High	*c*.20%	4.7	5.9	7.2	10.0	10.0	10.7	12.4	15.0	13.7
*iii*	Hybrid	Normal	*c*.80%	3.4	5.0	5.6	5.8	6.4	7.2	11.7	20.7	21.9
*iv*	Hybrid	High	*c*.20%	6.2	8.7	11.2	12.0	13.0	14.6	24.3	37.9	42.6

Shares are given in percentages and calculated respectively within the c.80% and c.20% distribution.

The relative shares were used in the following comparisons:

(a) *Comparison of the shares of gold and hybrid open access articles on the normal level (row no. i and row no. iii)*

The comparison of row no. *i* and no. *iii* in Table 3 shows that in the pre-PAR period, 2013–2018, researchers chose gold open access journals on the normal level on average 4.8 times more often than hybrid open access journals on the normal level. The PAR deals lowered that ratio, despite steady overall growth in the share of gold open access articles. In 2021, gold open access journals were chosen 1.9 times more than hybrid journals. Researchers chose gold open access publications more than hybrid open access publications throughout the period.

(b) *Comparison of the shares of gold and hybrid open access articles on the high level (row no. ii and row no. iv)*

The comparison of row no. *ii* and no. *vi* in Table 3 shows the opposite effect for articles on the high level. In the pre-PAR period 2013–2018, researchers chose hybrid open access journals on average 1.4 times more than gold open access journals on the high level. In 2021, hybrid open access journals were chosen 3.1 times more than gold open access journals. Researchers chose hybrid open access publications more often than gold open access publications on a high level throughout the period.

(c) *Comparison of the distribution of gold open access articles on the high and normal levels (row no. i and row no. ii)*

The comparison of row no. *i* and no. *ii* in Table 3 shows that the preference for submitting to gold open access journals increased steadily throughout the period 2013–2021 on both levels, except for small decrease on level high in 2021. The relative share of gold open access articles was much lower on the high level than on the normal level. The relative choice of gold open access journals on the normal level ranged from 4.1 times more often than on the high level in 2013 to three times more often than on the high level in 2021 (i.e., a slight decreasing rate). Researchers had a higher propensity to choose gold open access journals on a normal level than on a high level throughout the period.

(d) *Comparison of the distribution of hybrid open access articles on the high and normal levels (row no. ii and row no. iv)*

The comparison of row no. *ii* and no. *iv* in Table 3 shows the opposite effect of what was found in the case of gold articles. When researchers chose journals on a high level, the relative share of articles published hybrid was higher than the relative share of articles on the normal level. Choices on the high level were in the range of 1.7–2.1 times more often than choices on the normal level. Researchers had a higher propensity to choose hybrid open access publication on the high level than on the normal level throughout the period.

The distribution of hybrid open access articles between the high and normal levels may have been influenced by the introduction of PAR deals. To investigate this, the distribution between PAR hybrid and non-PAR hybrid articles on the high and normal levels was calculated separately for 2020 and 2021.

Table 4 shows that in 2020, non-PAR hybrid open access publication was chosen two times more often on the high level than on the normal level, while choices of PAR hybrid on the high level were 1.7 higher than on the normal level. The corresponding numbers for 2021 were 2 and 1.9, respectively.

**Table 4:** Shares of hybrid open access articles on the normal level compared with hybrid open access articles on the high level in 2020 and 2021.

	**2020**	**2021**
Non-PAR hybrid open access articles – normal level	7.8	9.4
Non-PAR hybrid open access articles – high level	15.9	19
PAR hybrid open access articles – normal level	12.9	12.4
PAR hybrid open access articles – high level	22	23.5

Shares are given in percentages and calculated respectively within the c.80 and c.20% distribution.

Viewed separately, PAR hybrid articles largely followed the same distribution as non-PAR hybrid articles, which also was stable throughout the period 2013–2021.

### RQ3: How does the varying potential for relevant high ranking gold open access journals relate to immediate open access publishing within the different research areas and disciplines?

In the preceding section (RQ2), I have shown that researchers had a lower propensity to choose gold open access journals on the high level than on the normal level. This is likely connected to the number of options researchers have. Of 28,319 eligible journals in 2021, 2193 were assigned to the high level and only 113 of those were gold open access journals.

**Figure 9:**
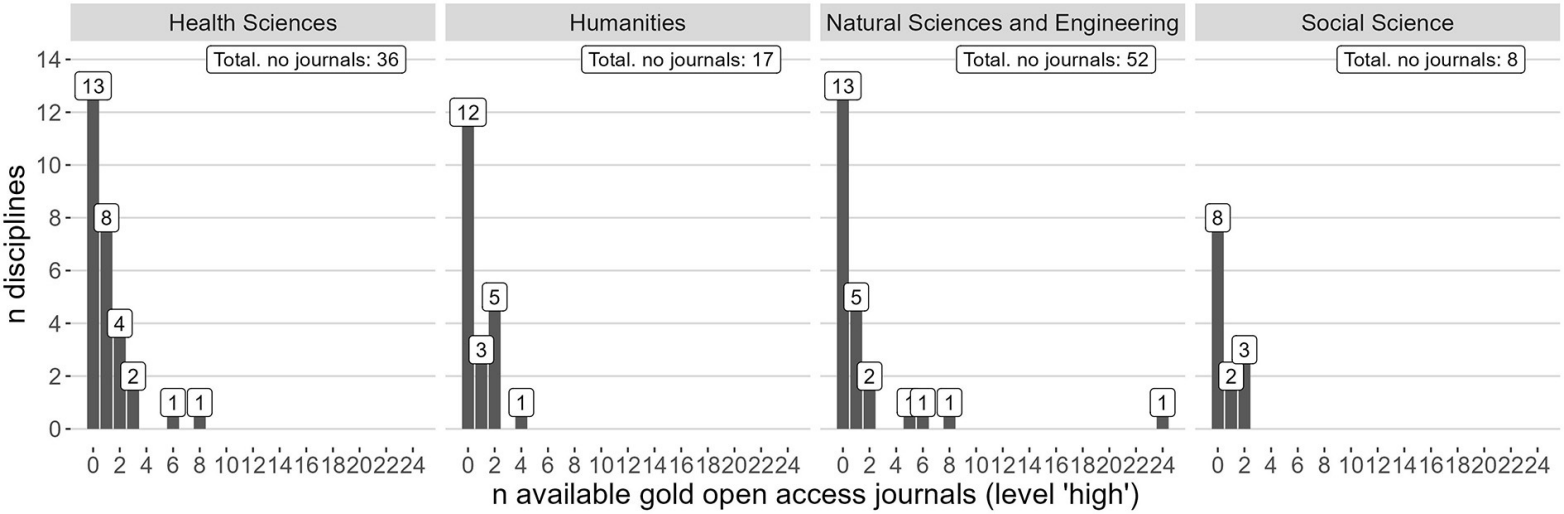
Frequency distribution of disciplines (*y*-axis) by the number of available gold open access journals on the high level (*x*-axis).

Figure 9 shows the frequency distribution of the 113 gold open access journals on the high level, by the number of disciplines within each area (*y*-axis) and the number of available journals (*x*-axis). For example, in the humanities, 12 disciplines were without any gold open access journals on level high, three disciplines had one gold open access journal on the high level at their disposal, and further five disciplines had two journals available, etc.

Figure 9 shows that 13 out of 29 disciplines within health sciences, 12 out of 21 disciplines within the humanities, 13 out of 24 disciplines in the natural sciences, and eight out of 13 disciplines within the social sciences were not represented by gold open access journals on the high level. In total, 46 out of 87 disciplines were without this option. Of the remaining 41 disciplines with gold open access options on level high, only nine disciplines had more than two journals. The number of gold open access journals on the normal level was more favourable in all research areas, with the average (rounded) number of options within each discipline being 44 in the health sciences, 38 in the humanities, 60 in the natural sciences, and 84 in the social sciences.

The distributions in Figure 9 only partially give a correct overview of the options a researcher has; this is due to the classification policy that all journals are allocated to a single discipline regardless of their interdisciplinary or multidisciplinary character. These journals are also part of the distribution in Figure 9.

Mega-journals can cover several disciplines, although most of these are placed on the normal level. It can also be relevant for researchers to publish in journals in neighbouring or related fields. No unambiguous method for controlling for such effects was found during the study. However, it was found that of the 94 journals on level high with articles published in 2020, 50 journals covered more than one discipline in the *All Science Journal Classification* (ASJC) system in Scopus. Of these, 11 journals had more than 10 publications in 2020, suggesting that ambiguous disciplinary classification is unlikely to alter the above result considerably.

To answer RQ3, the results from Figure 9 were used to split the production of articles into two groups by

- disciplines (within each of four research areas) with the option of gold open access journals on the high level (in a given year).
- disciplines (within each of four research areas) without the option of gold open access journals on the high level (in a given year).

This was achievable since each article in the dataset contained information about the journal's level at the time of publication and thus made it possible to reconstruct which disciplines had gold open access articles on the high level in the period 2013–2021. The resulting time series was used to investigate whether the two groups differed in

(1) gold open access publishing on the normal level and/or
(2) hybrid publishing on level high.

**Figure 10:**
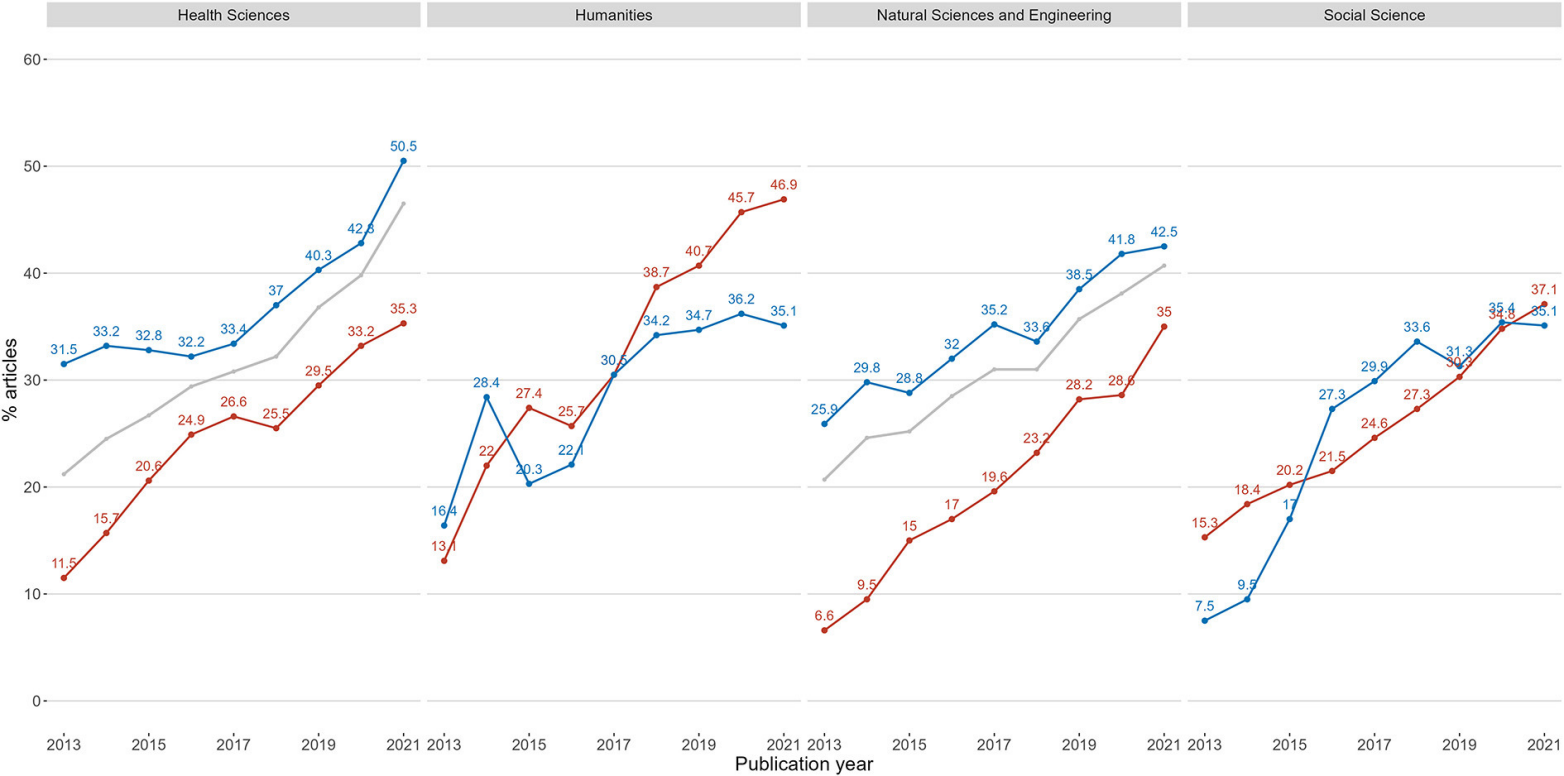
Shares of gold open access articles on the normal level for two groups of disciplines. Blue line represents gold publishing on level normal by disciplines with the option of gold open access journals on the high level, disciplines without this option is represented by the red line. For reference, the grey line represents the share of all gold articles on level normal regardless of the group. The plot is separated on the four main research areas.

To investigate the first possibility, the two groups were compared in Figure 10, which presents the shares of published articles in gold open access journals on the normal level (of *c*. 80% of totals). The shares contain all published articles in each group added together, which gives more weight to disciplines with a high rate of production. The analysis accounted for natural changes in the data, such as journals moving between levels, and consequently the incidental case that a discipline would be without gold open access journals on level high 1 year. Figure 10 also includes a grey line indicating the share of all gold articles on level normal regardless of groups, in the two research areas where there is an observable difference.

Figure 10 shows that in both the health and natural sciences, the gold publishing rate on level normal was consistently higher throughout the period for the group of disciplines which also had gold open access journals on the high level as an option.

The patterns in the humanities and the social sciences are less distinct, although the group without gold journals on level high in the humanities increased their share of gold articles from 2018 onward more than the group with gold journals on level high.

**Figure 11:**
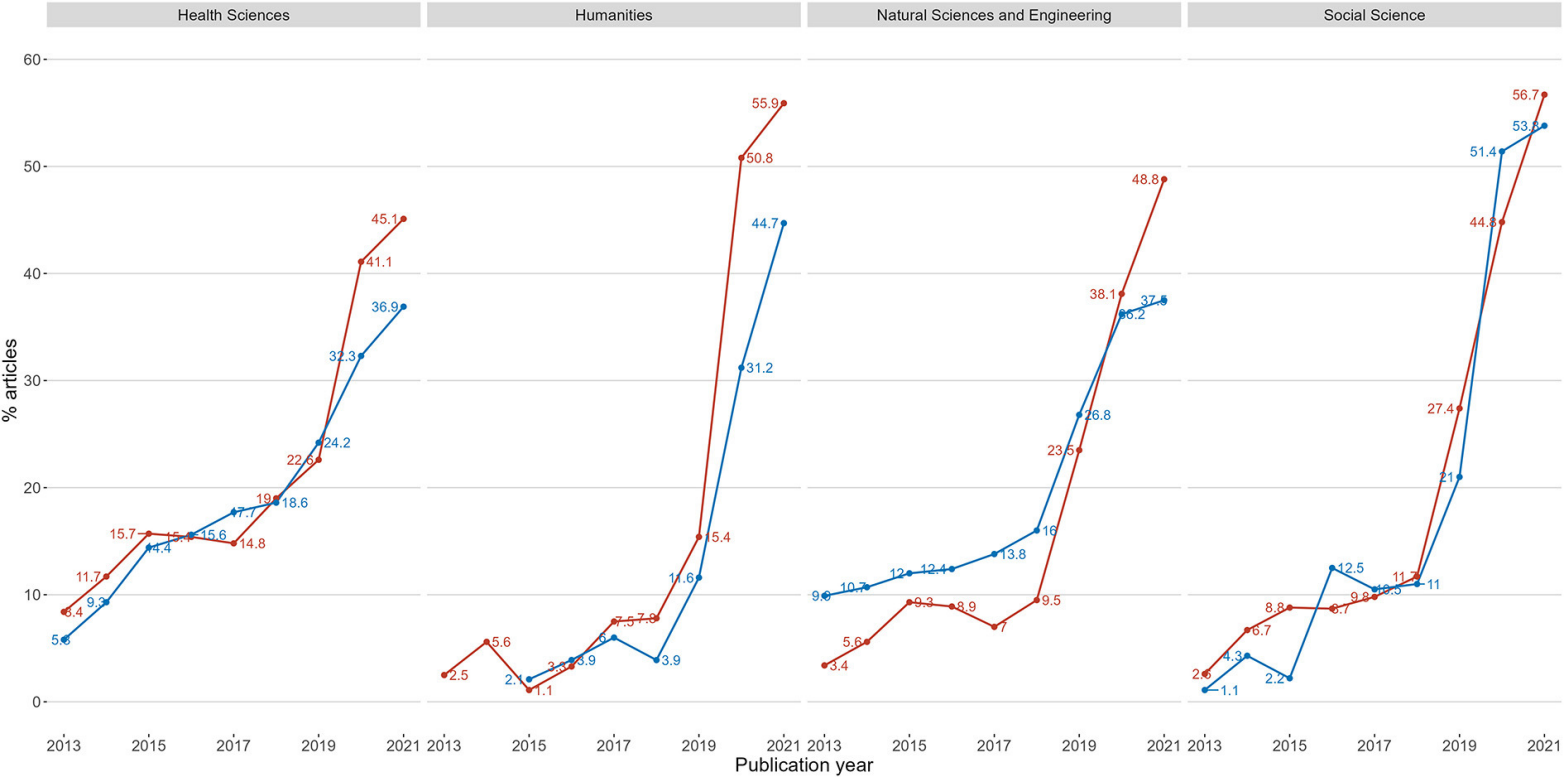
Shares of hybrid open access articles on level high for two groups of disciplines. Blue line represents hybrid publishing on level high by disciplines with the option of gold open access journals on the high level, disciplines without this option is represented by the red line. The plot is separated on the four main research areas.

Finally, the study investigated the second possibility of whether the absence of gold open access journals on a high level could drive publishing towards hybrid open access on level high. Figure 11 compares the two groups and their shares in hybrid open access articles on the high level (of *c*. 20% of totals). As in Figure 10, the yearly shares contain all published articles in the group added together, with more weight given to disciplines with high production rate.

Figure 11 shows a large increase in the uptake of hybrid on level high for both groups in all research areas after the introduction of PAR deals in 2019. The plot also indicates that disciplines without gold open access journals on the high level (red lines), except in the social sciences, published more hybrid on level high than the other group (blue line) after the introduction of PAR deals. In the social sciences, the two groups largely follow the same pattern even after the introduction of PAR deals.

There is however little to suggest that the lack of gold open access journals on level high, has had any impact on publishing in hybrid venues before the introduction of PAR deals.

Both groups in both the social sciences and the humanities had low levels of hybrid before the PAR deals in 2019, but higher levels of hybrid than both the health and natural sciences after the introduction of PAR deals.

### Summary

The share of hybrid open access articles increased slowly, from 4.1% in 2013 to 8.9% in 2018. With the introduction of transformative deals in 2019, there was a significant boost in the share of hybrid open access articles, which rose to 27.1% in 2021. PAR deal hybrid articles came in addition to non-PAR deal hybrid articles and seem to have replaced a sizeable share of green open access. It is unclear whether PAR deals have increased the overall share of open access.

Expressed in relative terms, researchers' propensity to choose the hybrid option doubled when they chose journals ranked on level high compared to journals on the normal level. Even though PAR deals boosted the overall share of hybrid open access, this distribution between levels did not change.

Conversely, researchers' propensity to choose gold open access journals decreased when they chose journals on level high compared to journals on a normal level. The relative share of gold open access articles on the normal level was three times higher than at the high level in 2021. The relative drop in gold open access on the high level corresponded with the overall low coverage of gold open access journals on the high level; 46 out of 87 disciplines were not represented with gold open access journals on level high in 2021.

The disciplines within the health sciences and the natural sciences without gold open access journals on the high level also published fewer gold open access articles on the normal level compared to the disciplines with this option. The pattern was consistent throughout the period. There was no similar pattern found in the humanities and social science.

The groups of disciplines in the health sciences, the natural sciences, and the humanities without gold open access journals on level high, also published more hybrid articles on level high than the group without that option, but only after the introduction of PAR deals. With only two full years of PAR deals (2020 and 2021) in operation, this is a conclusion drawn with some caution. However, in all research areas, the hybrid publishing on level high was boosted by the PAR deals.

## Discussion

The discussion will be organised in the same order as the research questions, starting with initial findings relevant to the research questions, the influence of PAR deals, followed by the relationship between journal ranking and immediate open access publishing before ending with disciplinary differences and limitations of the study. The article will then conclude with policy considerations and avenues for further research.

First, it should be noted that even if this study is of a descriptive nature and not set up as a correlation study, it has produced quantitative findings in a “controlled bibliographical space” on the country level, with the strength of a near complete national record over eight years. This has avoided known challenges connected to both coverage and baselines of articles (Huang et al., 2020a; Pölönen et al., 2020) and their open access modality (Else, 2018; Robinson-Garcia et al., 2020).

Initial and relevant findings for the research questions are that the share of gold open access on average was three to four times larger than hybrid articles in the period 2013–2018, up until the introduction of PAR deals in 2019. The larger uptake of gold open access may at first glance seem at odds with the idea that legacy journals generally are more prestigious than gold open access journals (e.g. Björk, 2013; Laakso and Björk, 2021). However, while the choice of gold open access journals necessarily accommodates immediate open access, the choice of hybrid journals may also result in an article being published with green open access or even closed. This choice is connected to the presence and level of APCs (Tenopir et al., 2017) and hybrid articles are considerably more expensive than gold articles, not financially supported by funds, and discouraged by policies. These factors partially explain why gold open access journals had a larger uptake than hybrid open access in the period 2013–2018, which also is consistent with previous prevalence studies (Piwowar et al., 2018).

The distribution changed with the introduction of PAR deals. Overall, the share of hybrid in 2021 (27.1%) approaches the same share as gold open access (34.4%). The boost in hybrid open access with the PAR deals can be explained by easy and cost-free “ticking of the right boxes” when researchers submit their work to the journal of choice. Hybrid also offers more attractive terms than green open access. The publisher's PDF becomes immediately open and there is no need for the cumbersome act of depositing a previous version of the article in a repository. The large uptake of PAR hybrid open access is likely connected to a corresponding drop in the share of green open access in the period 2019–2021 and suggests that PAR deals have at least to some degree replaced one modality of open access with another. With only 1 year of data (2021), it would be premature to conclude that PAR deals have not added to the total level of open access, although it is clear that if the share of deposited 2021-articles (after embargos have expired) end up at the same level as in 2020 and 2019 (~9%), the total level of open access in 2021 will have levelled out, if not slightly decreased.

Publish-and-read hybrid open access articles mainly came in addition to non-PAR hybrid articles instead of replacing them. This finding is somewhat unexpected but was confirmed by two procedures showing there was little change in the overall publishing rates in journals outside PAR deals and that the publication rate for non-PAR hybrid articles largely was the same for all Norwegian universities collectively. Despite the two confirming procedures, there is a possibility that the study was unable to control for: non-PAR hybrid articles in Norway could benefit from PAR deals in other countries where Norwegian researchers are engaged in the co-authorship of articles. This could partly compensate for any PAR hybrid replacement. Whether there is such an effect is a subject for future research.

A central finding is how journal ranking relates to researchers' choices of gold open-access journals. Against the backdrop of an increase in both hybrid and gold access articles, there was a consistently lower propensity for researchers to choose gold open access journals on a high level compared to the normal level. This finding should not be confused with the perceived general less prestigious role of gold open access journals in journal hierarchies previously reported in the literature (e.g., Laakso and Björk, 2021), but is an effect linked to the lack of gold open access journals on the high level. This finding is also consistent with previous studies of researchers' attitudes towards open access and journal ranks (e.g., Togia and Korobili, 2014).

It is more challenging to explain the higher propensity to choose hybrid open access when choosing journals on a high level. Although studies find that paying APCs for publication is a barrier to immediate open access, this has mainly been investigated in the context of gold open access publishing (e.g., Tenopir et al., 2017) and does not compare the same type of open access modality and the influence of journal ranks.

Therefore, a credible explanation could be that despite the lack of support from centrally handled funds and the discouragement in policies, researchers are more willing to pay for hybrid open access articles in journals of higher rank. However, the result that the distribution of articles on the normal and high level was largely the same for hybrid articles in PAR deal as for non-PAR hybrid articles, suggests that willingness to pay APCs is not the main cause for the effect. If APCs were a significant factor, articles in PAR deals would likely have had a different distribution between the levels than non-PAR hybrid articles. This would also be in agreement with previous studies showing that when APCs were paid centrally by institutional libraries or financers, expenditures were no longer a concern for researchers (Togia and Korobili, 2014). This effect is also a subject for future research.

Disciplinary differences in immediate open access publishing were investigated with respect to options of gold open access journals on the high level, where the absence of gold open access journals in 46 out of 87 disciplines obviously limits researchers' open access options if the journals' rank is of importance. The large increase in hybrid open access following the PAR deals was found in all four research areas. The disciplines within the health sciences and the natural sciences without gold open access journals on level high also had a lower uptake of gold open access on level normal, than the disciplines that have gold high-level gold open access journals at their disposal.

The study found some support for the idea that the lack of gold open access journals on level high could drive publishing into hybrid on the same level. This was found in the health sciences, the natural sciences, and the humanities, but only the introduction of PAR deals and thus based on a very short period of time. This is also consistent with previous findings that paying APCs is a barrier to immediate open access, which is relieved with central handling (Togia and Korobili, 2014).

The social sciences and the humanities, also previously found to have the lowest levels of open access (Severin et al., 2020), had a consistently low uptake of hybrid publishing on the highest level before the PAR deals in 2019, and the highest uptake after the introduction of PAR deals. The strong growth indicates that the PAR deals have satisfied a demand for the possibility to publish immediate open access. This is also consistent with previous studies reporting that project-based financing of immediate open access is difficult to obtain in the social sciences and the humanities (Severin et al., 2020).

This study has several limitations, the most obvious being the descriptive design and therefore the lack of causal explanations. However, the topic of open access as a research field is both relatively new and changing at a fast pace and only recently enabled by a more mature infrastructure. Descriptive analysis is in my view still needed to reveal structures, relationships, and associations, particularly in the case of PAR deals.

The framing of this study as the reward system in tension with choices of journals and immediately open access necessitates disregarding green open access. This decision is grounded in the cognitive distance between green open access and a researcher's choice of journal since green can be accommodated at any later point in time after publishing. However, removing green open access from the analysis has the drawback that any movements between hybrid and green that happen in the same set of journals, are lost. The relationship between hybrid and green open access is also an avenue for further research.

## Conclusion

In this study, I have investigated how researchers' immediate open access preferences were affected by journal ranking and the availability of attractive outlets, and how this, in turn, relates to publish-and-read (PAR) deals. The main theoretical argument in the study is that the academic journal has acquired a central position in the reward system of science (Merton, 1957), which influences researchers' choice of journals with regard to the submission of their articles (Nosek and Bar-Anan, 2012; Rushforth and de Rijcke, 2015; Gaston et al., 2020).

The main results are that gold open access publication correlates negatively with the Norwegian journal ranking; high journal rank correlates with relatively less gold open access publications. Hybrid open access correlates positively; high journal rank correlates with relatively more hybrid publications. Whilst PAR deals boost hybrid publishing considerably, journal ranking relates equally to both PAR and non-PAR hybrid uptake.

The findings confirm the tension between gold open access publishing and the reward system of science. This tension is connected to research assessment based on journal ranking and is still a barrier to be overcome for open access (European Commission, 2017; McKiernan et al., 2019; Saenen et al., 2019).

An important policy consideration is therefore the use of journal ranking in research assessment. Applying a journal ranking arguably leads to conflicting goals in Norwegian policies, which on the one hand promote open access and favour gold open access publication (Ministry of Education Research, 2017), but on the other hand reward publishing in journals based on their rank, without open access options or with less favourable options. The PAR deals smoothly with this conflict and makes available a range of popular journals, although only for researchers at participating institutions.

The Norwegian journal ranking system, partly based on impact factor, also corresponds with other established journal metrics. The implications of the study can therefore be extended to other systems that apply journal rankings in assessment procedures.

The finding that the Norwegian PAR hybrid comes in addition to the non-PAR hybrid, disregards the wider spread and uptake of PAR deals internationally. The ESAC-registry has registered 300 agreements that have been negotiated in over 30 countries as of March 2022, with the output more than tripled since the first PAR deal in 2019 (ESAC, n.d.). PAR deals in other countries likely influence the share of Norwegian hybrid outside the Norwegian PAR deals. Whether there is such an effect, is also a subject for future research. It is of high importance to monitor whether transformative agreements will eventually succeed in converting subscription-based journals at scale (Borrego et al., 2020). A point also to be considered by science policy, is that the popularity of hybrid in PAR deals likely comes at the expense of green open access. Likewise, whether PAR deals accommodate more open access or just different open access remains uncertain and is also a subject for further research.

The study also informs the broader picture of the transformative strategy as laid out by Plan S and Norwegian policies. If the transformation scheme proves successful and existing journals convert to gold open access, these will still hold a prominent position in the reward system of science with the risk of flawed research assessment, despite convincing arguments against the validity of journals as proxies for quality in research (DORA, 2012; Hicks et al., 2015). This would require revisions of research assessment procedures.

## Data availability statement

Publicly available datasets were analysed in this study. This data can be found at: https://doi.org/10.18710/TBXXCC.

## Author contributions

The author confirms being the sole contributor of this work and has approved it for publication.

## Funding

The PhD project is financed by the Research Council Norway (RCN-project: 272456) and is also a part of the Oslo Institute for Research on the Impact of Science (OSIRIS, RCN-project: 256240/O30).

## Conflict of interest

Author LW is a PhD candidate at the TIK-Centre at the University of Oslo while holding a position at Sikt, a governmental body reporting to the Ministry of Education and Research in Norway. This position is in the department that holds the responsibility of coordinating open access affairs in Norway.

## Publisher's note

All claims expressed in this article are solely those of the authors and do not necessarily represent those of their affiliated organizations, or those of the publisher, the editors and the reviewers. Any product that may be evaluated in this article, or claim that may be made by its manufacturer, is not guaranteed or endorsed by the publisher.

## Footnotes

^1^Original text in Norwegian has been translated to English by the author of the study.

^2^This part of the guidelines has not yet been put into effect as of February 2022.

^3^Agreements were made with the publishers Elsevier, Wiley, Springer Nature, Cambridge University Press, Oxford University Press, Sage, De Gruyter, ACS (American Chemical Society), Taylor & Francis and RSC (Royal Society of Chemistry).
